# Acupuncture is an effective therapy for macular damage: A case report

**DOI:** 10.1097/MD.0000000000034735

**Published:** 2023-08-25

**Authors:** Qi Lu, Mengmeng Sun, Jinfeng Cao, Weizheng Wang, Haili Wang, Yu Gao, Ying Wang, Xiaole Guo, Weiwan Yang, Hongfeng Wang

**Affiliations:** a Department of Acupuncture and Tuina, Changchun University of Chinese Medicine, Changchun, China; b Changchun University of Chinese Medicine, Changchun, China; c Department of Ophthalmology, The Second Hospital of Jilin University, Changchun, China; d Department of Acupuncture and Tuina, The Third Affiliated Hospital of Changchun University of Chinese Medicine, Changchun, China.

**Keywords:** acupuncture, case report, local-distal acupoints, macular injury

## Abstract

**Rationale:**

Many factors can contribute to the development of macular injury, which results in vision loss as a result of a disease. Heredity, age, underlying eye illness, internal eye surgery, or eye trauma can all cause it. A safer alternative to current therapies for macular degeneration is urgently needed since they all induce ocular irritation and postoperative recurrence as well as a host of other adverse effects.

**Patient concerns:**

A 12-year-old girl was the patient. A laser pen burnt her right eye. There was a spot and a shadow in the middle of her right eye’s visual field.

**Diagnoses:**

Macular degeneration.

**Interventions:**

Given the patient’s age, we opted out of medicine and instead used acupuncture as a symptomatic treatment.

**Outcomes:**

Two months after therapy concluded, optical coherence tomography result report indicate that the macula region of the right eye is better than it was previously. The corrected visual acuity of the right eye recovered from 0.25 to 1.0, and the clinical accompanying symptoms of the right eye disappeared.

**Lessons:**

No additional medication or surgical procedure was employed in this instance. We treated the macular damage with acupuncture, which relieved the patient’s clinical symptoms and had no adverse effects. This demonstrates that acupuncture may be beneficial in treating ophthalmopathy in this direction.

## 1. Introduction

Macular disease is a condition in which macular lesions develop in the retina, resulting in clinical symptoms such as blurred vision, distorted vision, central scotoma, or loss of central vision. Scotoma development is connected with damage or death of photoreceptor cells, retinal pigment epithelial cells, and choroidal endothelial cells, and the macula is vulnerable to oxidative and ischemia stress, with oxidative stress being associated with macular injury.^[1,2]^ At present, it is believed that the occurrence of the disease is related to many factors, such as: hereditary lesions,^[3]^ age-related macular degeneration,^[4]^ postoperative eye disease and trauma in this case factors.^[5]^ Macular degeneration has a variable prognosis. Severe instances may result in irreparable vision damage or possibly blindness. According to statistics,^[4,6,7]^ over 900 million individuals would suffer from eye problems by 2050, as a result, we should prioritize macular degeneration prevention and treatment. Macular degeneration is being treated with medicine, surgery, and other technologies. There are some disagreements and reservations about complicated operation, secondary adverse effects, dosage, and the synergistic effect of combined drug use. Surgical treatment is typically chosen when medication management is insufficient.^[8–10]^ While good surgical outcomes can last around 2 years, they cannot prevent macular atrophy and vision loss. Each of the treatments listed above has a number of pros and downsides, which is why it is critical to seek for a successful treatment approach that is also safe and has a low risk of adverse effects.

Acupuncture has been shown to safely increase the retinal blood oxygen balance and local microcirculation in order to aid in the recovery of eye diseases,^[11]^ as well as alter the content of relevant metabolites and the production of certain eye disease-related substances,^[12,13]^ indicating that acupuncture may have a therapeutic impact on macular disorders.

We present a case of macular injury induced by a laser burn that was successfully cured with acupuncture.

## 2. Case report

### 2.1. Clinical presentation

**Figure 1.**
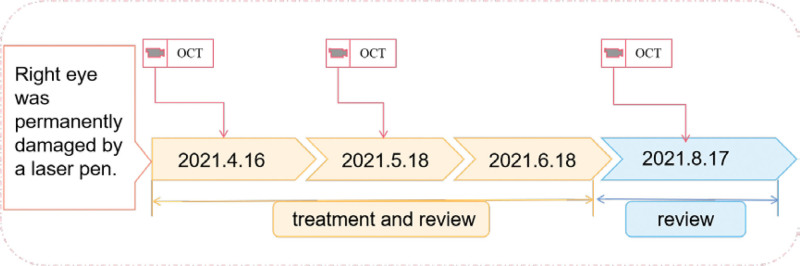
Timeline of clinical and procedural data.

The timeline with clinical and procedural data is shown in Figure 1.

A 12-year-old girl was the patient. Her right eye was permanently damaged by a laser pen. The spot and shadow emerged in the middle of her right eye’s visual field. She sees a doctor for acupuncture therapy because, in addition to the aforesaid symptoms, she experienced eye tiredness, worsened dryness, and eye discomfort. The patient had a free previous medical history. There are no further eye abnormalities, no prior trauma or surgery, and no family genetic condition.

**Figure 2.**
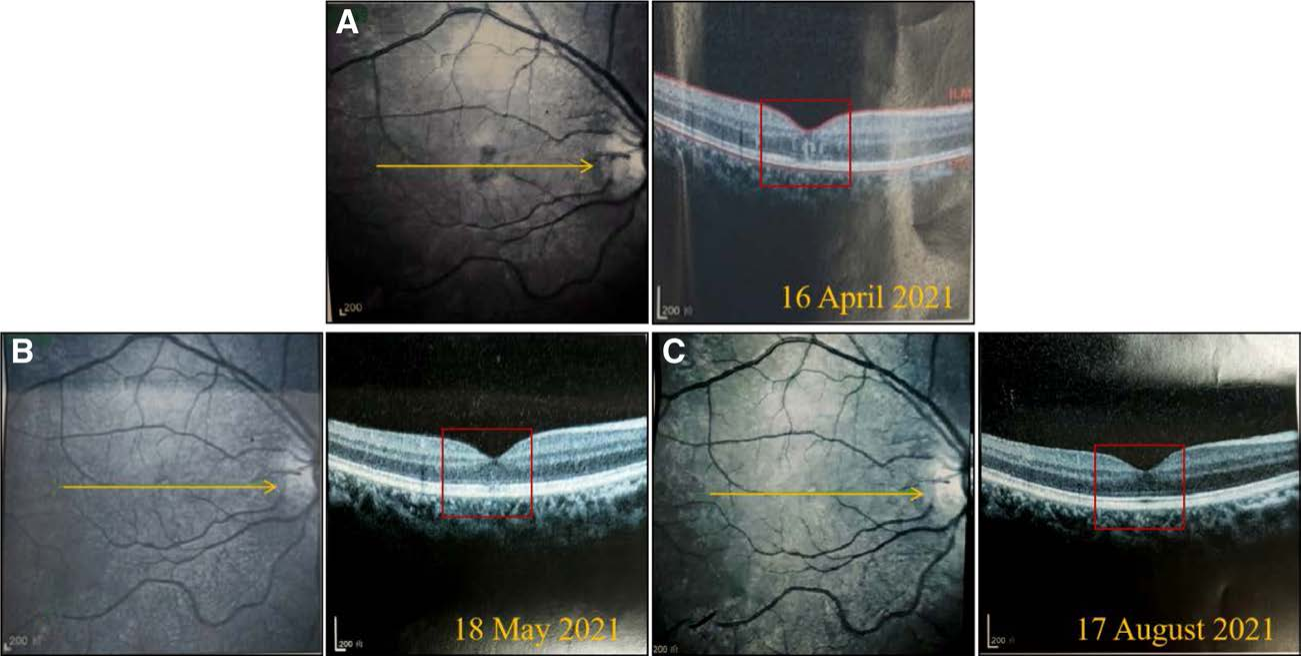
Results of The Right Eyes OCT. The yellow arrows denote the scan’s position and direction. (A) On April 16, 2021. The red square depicts a tiny region of significant reflection in the macula. (B) On May 18, 2021 the right eye OCT was reexamined. The red square indicated the presence of a tiny region of moderate reflection in the macula’s retina. (C) On August 17, 2021. The red square indicates that the macula region inside the omental reflection signal area has improved. OCT = optical coherence tomography.

Physical examination: Ophthalmic examination: best-corrected visual acuity: Right = 0.25, Left = 0.8; Optical coherence tomography (OCT): In the macula of the right eye, a tiny, patchy, mild reflection region is visible (Fig. 2A). Left eye normal. Finally, it was established that the patient suffered from macular degeneration.

### 2.2. Interventional procedure

Given the patient’s age, we opted out of medicine and instead used acupuncture as a symptomatic treatment. For 30 minutes once a day, acupuncture was used as a therapeutic at specific acupoints (Table 1).

**Table 1:** Acupuncture point.

Table 1 Local acupoints		Distal acupoints	Special acupoints
The brain	The eye		
Visual area			
Bai Hui (DU20) Shen Ting (DU24)	Yin Tang (DU29)		
Feng Chi (GB20)	Yang Bai (GB14)		Guang Ming (GB37)
	Tai Yang (EX-HN5)Yu Yao (EX-HN4)		
	Cuan Zhu (BL2)		
	Si Zhu Kong (SJ23)	Wai Guan (SJ5)	
	Si Bai (ST2) Xia Guan (ST7)	Zu San Li (ST36)	
		He Gu (LI14)	
		Tai Chong (LR3)	

After 1 week of acupuncture therapy, the patient reported significant alleviation from ocular discomfort symptoms, including the disappearance of dry symptoms, a decrease in the frequency and severity of pain, and impaired vision. After 4 weeks of continuing therapy, the girl stated that the discomfort in her eyes had subsided, the imperfections in her field of vision had vanished, and her eye movements were unaffected. The results of the first review: Best-corrected visual acuity: Right = 0.8, Left = 1.0; OCT: There were small patches of medium reflected signal in the retina of the macular area of the right eye that disappeared obviously. Comparison between the 2 results showed that the macular area of the right eye was better than before (Fig. 2B).

Her symptoms progressively improved, continue using the following approaches to maintain efficacy for another 4 weeks, for a total of 8 weeks of therapy. Following therapy, the patient’s clinical symptoms totally resolve. It is recommended that the patient discontinue therapy, relax during ordinary time, avoid excessive eye tiredness, and advise the patient to reassess after 8 weeks.

### 2.3. Outcome and follow-up

On August 17, 2021, 2 months after therapy concluded, the patient was contacted via telephone. Since then, the patient has had no more treatment, and the therapeutic benefits of acupuncture have remained excellent, with no return of clinical problems. OCT result report (Fig. 2C) indicate that the macula region of the right eye is better than it was previously. Thus far, OCT imaging of retinal damage in individuals with this condition has been superior than the previous period. The corrected visual acuity of the right eye recovered from 0.25 to 1.0, the visual acuity recovered, and the clinical accompanying symptoms such as pain and easy fatigue of the right eye disappeared.

## 3. Discussion

Western medicine have a no unified plan for the macular injury caused by laser burn in this case, but gives symptomatic treatment according to the condition. On the 1 hand, many drugs in the treatment are cumbersome and have large side effects, and even induce other eye diseases. On the other hand, currently there is no particularly effective treatment for some mild diseases in Western medicine, while acupuncture has good clinical efficacy and no side effects. Here we discuss the feasibility of traditional Chinese medicine (TCM) acupuncture in treating eye diseases.

TCM has a unique meridians theory to support the pathological changes and dysfunction of local tissues or organs. The twelve meridians are the main body of the meridians system. They are respectively affiliated to the twelve viscera. Twelve meridians constitute a cycle, such as endless channel system, to maintain the function of the material basis through the meridians can be inward to the Zang-Fu organs, outward to the muscle surface, running to the body of each part. The eye or around the eye is related to multiple meridians. We believe that local tissue damage caused by burns will cause periocular meridians obstruction, that is, blood circulation and material circulation in the local tissues of the eye are obstructed or even blocked, thus showing symptoms such as impaired eye function and decreased vision. It can be seen from the above, TCM from the whole point of view, adjust and improve the operation of the meridians related to the body so that the local veins unobstructed and other aspects of the treatment of eye diseases. The eyes are closely related to the spleen, stomach, liver and other organs. The spleen and stomach digest and absorb food, transforming to bring the nutrition needed by the body. The body can only make the blood fill with sufficient nutrition, so as to provide continuous power for the operation of the physiological function of the eyes. In addition to the spleen and stomach, liver is more closely related to the eye, and relevant studies have confirmed that there is indeed a connection between liver and eye pathway.^[14]^ Closely linked meridians in the body each viscera and maintaining the normal operation, the physiological function of liver meridian courses associated with the eye, liver stores blood, spleen and blood, spleen and stomach and liver are the generation, the source of nutrition of qi and blood, and the dynamic operation, sufficient blood physiological and function to maintain normal work, so that the eye so treat eye disease should pay attention to the related regulation of meridians. Acupuncture is a traditional clinical application under the guidance of the theory of meridians and acupoints. We can stimulate the acupuncture points on the meridians to strengthen the connection between the 3, and achieve the purpose of treating diseases by acupuncture points to regulate the meridians. The success of this case is inseparable from reasonable acupoint selection. Since local acupoints and distal acupoints may belong to different meridians, synergic effects can be achieved by using them together.^[15]^ Acupoint selection can be divided into local acupoint selection, distal acupoint selection and empirical acupoint selection. Proximal acupoint selection is to select similar acupoints for treatment according to the location of the disease, distal acupoint selection is to select the far meridians and collaterals related to the disease, and experiential acupoints are acupuncture points that have special therapeutic effects for a certain disease. In accordance with the principle of acupoint selection, the disease is more closely related to the Zang-Fu organs of the far part of ST36, LI4, LR3 (they can contact the meridians of the relevant Zang-Fu organs and have a representative role in acupoints.) and other acupoints, and the local eye, head acupoints mainly, Such as DU29, EX-HN4, EX-HN5, GB14, BL2, SJ23, ST2, ST7, and the visual area of the eye in the cerebral cortex reflex area. In this case, the selection of points focuses on the improvement of the overall balance of meridians and organs. Relative to the disease location far and near with acupoint selection, not only can adjust the corresponding meridians usually associated with the eye, but also can enhance the overall contact between the eye and the body, inside and outside, and improve the overall environment of health, especially in the process of focusing on the improvement and treatment of the local conditions of eye lesions, In addition, with the GB37 for the eye disease itself (GB37 is the treatment of eye disease experience effect point, Chinese medicine in the name of the point from the light through the meaning of light, meaning can be bright), through stimulating the meridians to connect the whole inside and outside and local to achieve the purpose of treating the disease.

In this case, the neural uplift in the macular area and signal changes in the IS/OS layer indicate abnormal conduction connections between the inner and outer segments of the retinal photoreceptors. This is also the main cause of visual acuity loss caused by the influence of shadow in the center of the eye. As mentioned above, the appearance of shadow is associated with photoreceptor cells and cell damage or death. There is evidence that acupuncture visual signal intensity and the related points,^[16–19]^ it can enhance brain regions of the brain’s visual cortex between specific benign adjustment, and acupuncture can stimulate the neurons around eyes, acupoint is located on the main beam of neurons, more have adjust the function of peripheral and central nervous pathway activity, also can stimulate self-adjusting process. The position of the visual area corresponds to the visual center, and the fibers of the macular area project to the position of 1/3 of the posterior part of the visual area, so acupuncture in the visual area can strengthen the regulation of local nerve conduction and the stimulation of functional areas. Combined with the theory of TCM, we believe that the improvement of eye diseases, the damage of repair and the inhibition of disease process are driven by the abundant nutrition supply, that is, the blood supply is mainly relied on. The eye blood circulation supply mainly comes from the eye artery, and the eye artery filling depends on the branches of the internal carotid artery, which is closely linked with multiple optic nerves. Therefore, acupuncture of the head, neck and local relevant acupoints can improve the head and face blood circulation, so as to improve the blood supply of the eye and optic nerve and enhance tissue metabolism. Eye local acupoints has a wealth of nerves and blood vessels, the same acupuncture this hole can obviously improve the local blood circulation around eyes, achieve the purpose of flow around the eyes veins, and thus enhanced the oxygen utilization rate of nerve cells, promote the metabolism of oxygen stimulate the peripheral nerves, improve nerve function, and can promote the damaged nerve repair and regeneration. Some doctors have successfully improved eye diseases by acupuncture at local relevant acupoints.^[20,21]^ We also expect local acupuncture points such as GB20, GB14, EX-HN5, ex-HN4, BL2, SJ23, ST2, and ST7 to play a positive role in this regard. In addition to the proximal local acupoints playing a positive role in the curative effect, we also found the mechanism of the distal acupoints in the treatment of eye diseases. Acupuncture of LI4 in the distal part can promote the synthesis and secretion of relevant neurotransmitters,^[22]^ thus strengthening the nutrition and protection of neurons, repairing conduction abnormalities, reversing visual impairment, and thus improving the disorder of visual environment. In the far department find the LR3 can promote the brain areas associated with visual acupuncture can activate blood flow and activation occipital visual cortex,^[23]^ and thus makes coefficient increases blood flow to the eye, can improve local microcirculation, promote ocular blood oxygen balance and improve visual function, adjusting the eye of the nervous system and blood supply situation to improve the eye disease. Generally, the 2 are regarded as a pair, and some studies have shown that the combination of the 2 can regulate the related expression of visual cortex.^[22–24]^ At the same time, there is evidence that acupuncture can improve ocular lesions by inhibiting the activity of retinal microglia and blocking the apoptosis process of photoreceptor cells. In general, by improving the blood circulation function of the brain, the brain and ocular nerve function can be improved, the local blood circulation state of the ocular nerve can be effectively improved, the cell damage can be improved to maintain the nervous system function, and the eye damage can be promoted to repair. The combination of the above points can regulate the overall state of the body and promote the occurrence of benign changes. Local focus on improving the microcirculation around the eye, blood oxygen balance for eye injury recovery from the internal can provide a steady power to lay a solid foundation. Based on the root of the disease, the treatment plan attaches importance to the overall regulation of the body and the improvement of the local disease, so the clinical effect is remarkable.

Although the case has achieved favorable clinical outcomes, we believe that the success is contingent upon the occurrence of several uncertainties: the patient is young, the body is still developing, and the patient possesses exceptional healing potential. And the patient is receiving prompt medical care and active medical intervention, which is also contributing to her rapid and complete recovery. Additionally, the laser pen unintentional damage is acute lesions, despite the fact that they are produced by macular burns and the current symptoms are rather visible, but patients with normal eye health save myopia, which is also critical for macular injury recovery, are excluded. Finally, the patient’s right eye vision decreased from 1.0 to 0.25 swiftly owing to the blockage of the field of vision shadow. Acupuncture therapy causes the shadow to vanish quickly, resulting in the patient’s outstanding vision recovery.

## 4. Conclusion

In conclusion, this case demonstrates solid clinical evidence for the use of acupuncture to treat ocular disorders, particularly macular injuries, and demonstrates a novel strategy to treating macular diseases. We can combine current medical resources with Western medicine to create a more optimal treatment approach.

## Acknowledgements

The authors would like to thank the patient and his guardians.

## Author contributions

**Conceptualization:** Mengmeng Sun.

**Methodology:** Weizheng Wang.

**Resources:** Hongfeng Wang, Jinfeng Cao.

**Supervision:** Haili Wang, Ying Wang.

**Writing – original draft:** Qi Lu, Yu Gao.

**Writing – review & editing:** Xiaole Guo, Weiwan Yang.
